# Antiproliferative Effect of the Isoquinoline Alkaloid Papaverine in Hepatocarcinoma HepG-2 Cells — Inhibition of Telomerase and Induction of Senescence

**DOI:** 10.3390/molecules190811846

**Published:** 2014-08-08

**Authors:** Sakineh Kazemi Noureini, Michael Wink

**Affiliations:** 1 Department of Biology, Faculty of Science, Hakim Sabzevari University, P.O. Box 397, Sabzevar, Iran; 2 Department of Biology, Institute of Pharmacy and Molecular Biotechnology, Heidelberg University, INF 364, 69120 Heidelberg, Germany

**Keywords:** papaverine, telomerase, immortality, senescence

## Abstract

Cancer cells are often immortal through up-regulation of the hTERT gene, which encodes the catalytic subunit of a special reverse transcriptase to overcome end-replication problem of chromosomes. This study demonstrates that papaverine, an isoquinoline alkaloid from the Papaveraceae, can overcome telomerase dependent immortality of HepG-2 cells that was used as a model of hepatocarcinoma. Although this alkaloid does not directly interact with telomeric sequences, papaverine inhibits telomerase through down-regulation of hTERT, which was analysed using thermal FRET and qRT-PCR, respectively. The IC_50_ values for the reduction of both telomerase activity and hTERT expression was 60 µM, while IC_50_ for cytotoxicity was 120 µM. Repeated treatments of the cells with very low non-toxic concentrations of papaverine resulted in growth arrest and strong reduction of population doublings after 40 days. This treatment induced senescent morphology in HepG-2 cells, which was evaluated by beta-galactosidase staining. Altogether, papaverine can be regarded as a promising model compound for drug design targeting cancer development.

## 1. Introduction

The most prominent characteristic of cancer cells is immortality, which is mainly due to the activity of telomerase, a ribonucleoprotein reverse transcriptase [1]. This enzyme compensates shortening of the ends of linear eukaryotic chromosomes that normally occurs in each cell division because of end-replication problem [2]. However, the critical role of telomerase has been demonstrated as telomere homeostasis. This enzyme is responsible for the immortality of cancer cells and is strongly over-expressed in about 90% of malignancies. Nevertheless, enzymatic component of telomerase, hTERT, plays a dual role in the regulation of telomere length. It regulates the optimal telomere length at each chromosomal end for efficient protection also in primary human cells, through shortening excessively long telomeres and elongating short telomeres simultaneously in one cell [3]. Telomere length plays critical roles in genomic stability, proliferation and differentiation of cells [4], somatic cell reprogramming [5] and mammalian gametogenesis [6]. On the other hand, telomeric dysfunction is well known to be involved in cellular senescence, genome instability and carcinogenesis [7,8].

**Figure 1:**
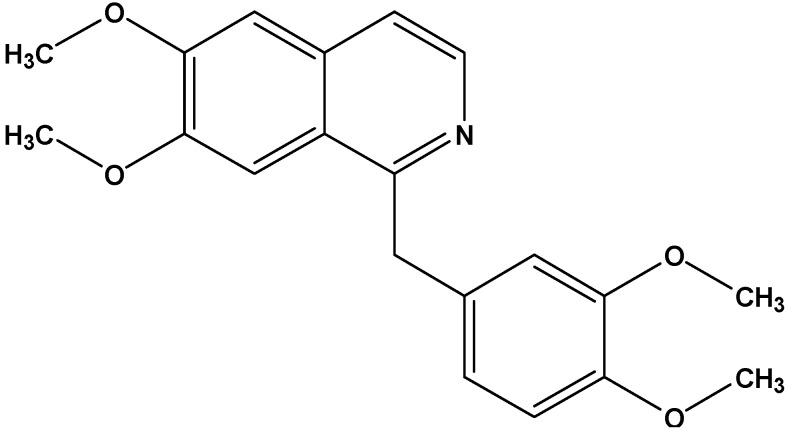
Papaverine (MW 339.40).

Therefore, one of the promising strategies in combatting cancer is a suppression of telomerase [9,10]. Cancer chemoprevention by natural products which interfere with telomerase offers excellent opportunities to reduce cancer risk. Screening potential telomerase inhibitors among plant secondary metabolites which exhibit a wide structural diversity may generate new valuable candidates for designing improved drugs. In an initial screening among several classes of different secondary plant metabolites the isoquinoline alkaloid papaverine (Figure 1) from the Papaveraceae, showed considerable reduction of cell growth in cancer cell culture models.

Papaverine is known to inhibit replication of human immunodeficiency virus (HIV) in H9 cell line and in peripheral blood mononuclear cell culture [11]. A papaverine derivative caroverine showed inhibition of VEGF and CoxII transcription in LT97 adenoma and SW480 carcinoma cell lines of colon cancer exposed to lipid peroxidation condition [12]. Papaverine acts as a non-selective phosphodiesterase inhibitor in mammals, increasing the amount of the secondary messengers cAMP and cGMP available for cell signalling [13]. In zebrafish embryo, papaverine increases cAMP levels in retinal-pigmented epithelium and inhibits melanocyte migration [14]. This compound is known to inhibit cell migration and delay zebrafish development, by suppressing processes that interact with the *kit-* signalling pathway [15]. However, the effect of papaverine on cancer cell proliferation is yet obscure, while other isoquinoline alkaloids, such sanguinarine [16] and chelidonine are potent anti-proliferative compounds. Chelidonine has already shown concomitant strong telomerase inhibitory and apoptosis induction effects [17].

Hepatocarcinoma is one of the most common types of malignancy worldwide, leading to >500,000 mortalities every year, with an overall 5-year survival rate of <10% [18]. About 90% of tumor samples are telomerase positive [19]. It has been shown that long telomeres are positively associated with the invasive capacity of hepatocarcinoma cells: The cells containing long telomeres have a high-level expression of invasion-promoting genes and a low-level expression of invasion-suppressing E-cadherin and therefore have a higher invasive capacity than those containing short telomeres [20].

In this study, we have focused on the antiproliferative activity of papaverine in the HepG-2 hepatocarcinoma cell culture model, especially by estimating telomerase activity and cell cycle control. We studied telomerase activity and the expression level of hTERT gene. We also show that papaverine does not intercalate telomeric DNA.

## 2. Results and Discussion

### 2.1. Papaverine Shows a Moderate Cytotoxicity in HepG-2

**Figure 2:**
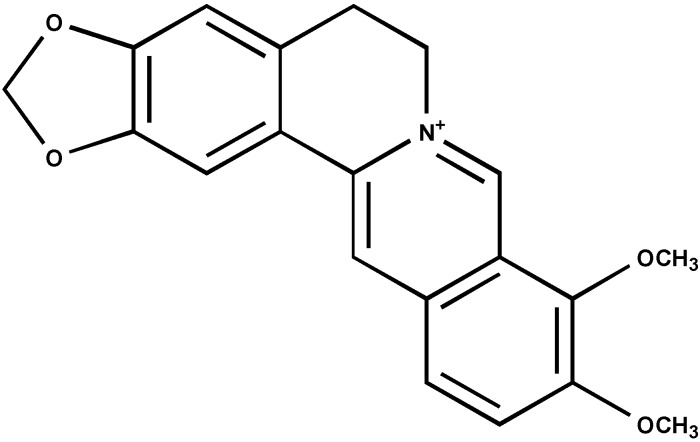
Berberine (MW 336.37).

**Figure 3:**
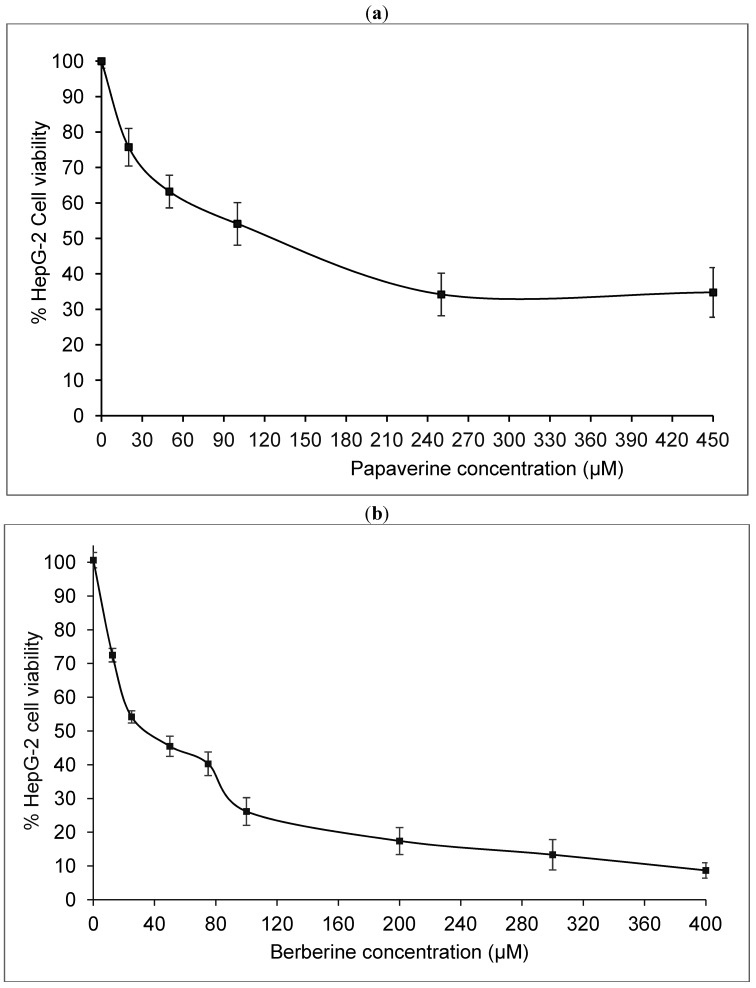
Cell viability after 48 h treatment of HepG2 cells with different concentrations of papaverine (**a**) and berberine (**b**). The curves represents mean values ± SD of three independent experiments, each in triplicates, *p* ≤ 0.05–0.02.

Cytotoxicity of papaverine and berberine (Figure 1 and Figure 2) in HepG2 cells was estimated using the MTT test which is based on measuring viability of cells under treatment in comparison with untreated controls. The dose response curve is illustrated in Figure 3a The IC_50_ value of papaverine is 120 µM after 48 h and thus much lower than those of berberine (Figure 3b) and chelidonine, known alkaloids with telomerase inhibitory activity, exhibiting IC_50_ values of 40 µM and 12.5 µM, respectively [17].

### 2.2. Telomerase Activity Is Reduced in Papaverine-Treated HepG-2 Cells

**Figure 4:**
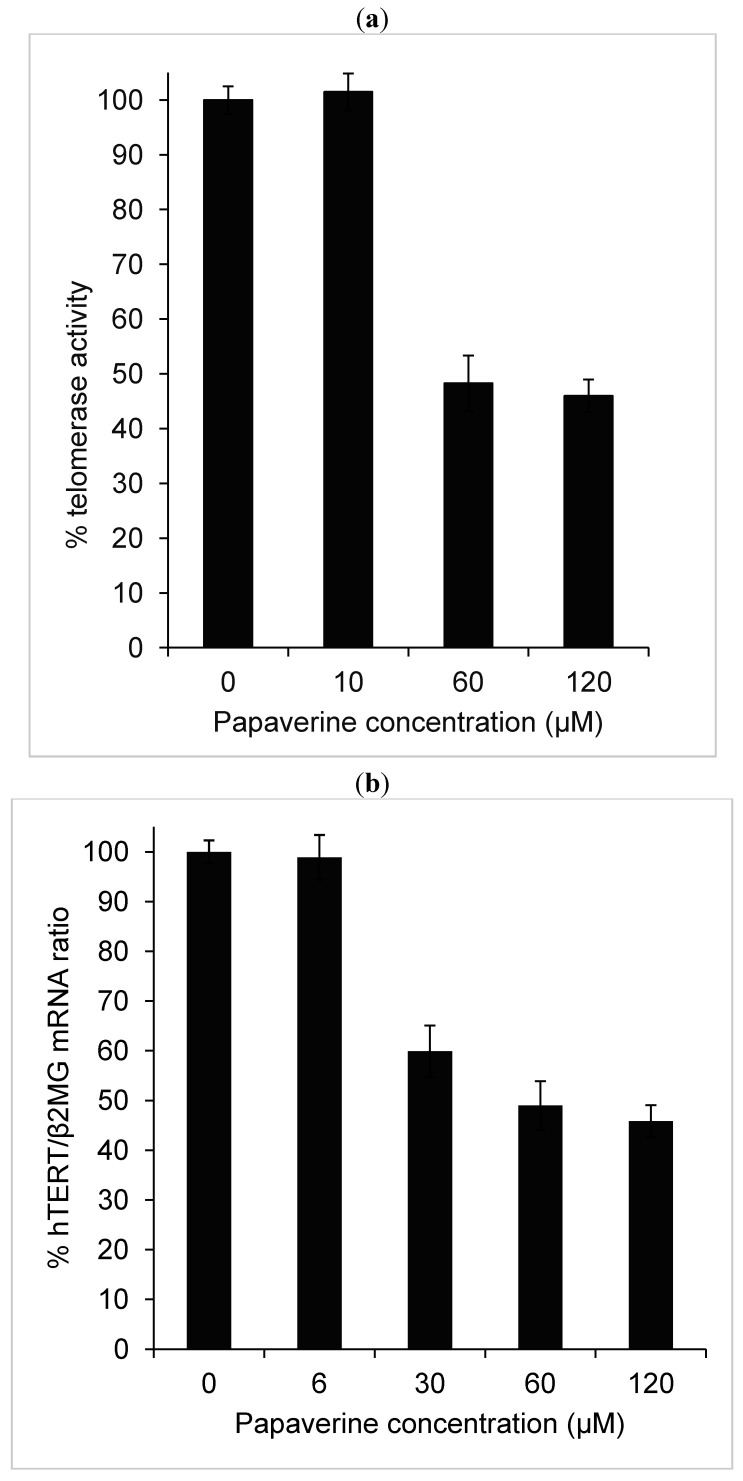
(**a**) Telomerase activity based on qTRAP assay and (**b**) hTERT mRNA level after 48 h treatment with papaverine. Beta-microglobulin mRNA was used to standardize the gene expression experiments. The measurements represent means ±SEM, *p* ≤ 0.01.

Quantitative telomere repeat amplification protocol that measures telomerase activity showed a strong decrease in telomerase activity after 48 h treatment with papaverine. Reduction of telomerase activity was apparent after treatment with papaverine concentrations higher than 10 µM. The highest effect was seen with 60 µM, which represents a substantial lower concentration than the corresponding IC_50_ value for cytotoxicity (Figure 4). Telomerase activity was reduced to less than 50% at a concentration of 60 µM. Berberine, a potent DNA intercalator, that reduces telomerase activity to 50% at 15 µM is much more powerful than papaverine. However, telomerase activity reduction in HepG-2 cells under treatment of papaverine and chelidonine (based on [17]) is similar.

### 2.3. Papaverine does not Directly Interact with Telomeric Sequences

**Figure 5:**
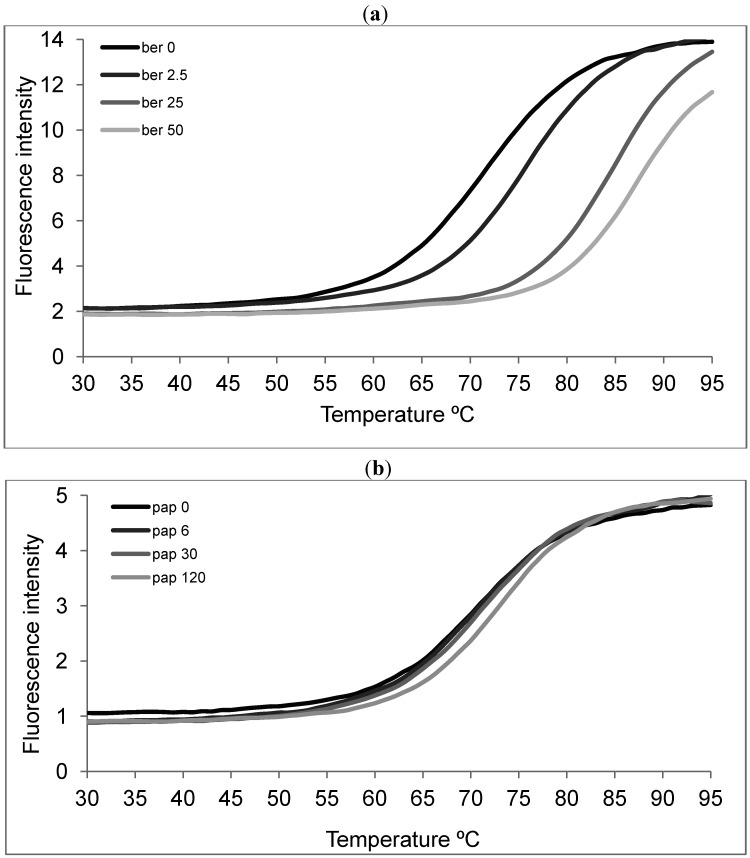
Thermal FRET analysis of F21T (0.25 µM), telomeric oligonucleotide in presence of increasing amounts of alkaloids. Fluorescence intensity versus temperature in presence of berberine (**a**: Black 0, dark gray 2.5, gray 25, light gray 50 µM) and papaverine (**b**: Black 0, dark gray 7.5, gray 30, light gray 120 µM) in potassium cacodylate buffer.

Strong telomerase inhibitors are often DNA intercalating drugs which can stabilize telomeric G-quadruplex structures. We have analysed whether papaverine has intercalating properties. Papaverine was incubated together with F21T, a synthetic oligonucleotide containing telomeric repeats. A typical ligand with G-quadruplex stabilizing characteristics will increase the melting temperature by at least 15 °C. In our experimental condition berberine the known alkaloid with telomeric G-quadruplex stabilizing effect increased T_m_ almost by 15.5 °C at a concentration of 50 µM, which is equal to about 200 equimolar to F21T (Figure 5a). Thermal FRET analysis however indicated that 120 µM papaverine (480 equimolar to F21T) induced only a small increase in T_m_ of F21T. Although these two alkaloid have very similar structures, berberine while having a more rigid structure than papaverine, contains a positive electric charge that can enhance its interaction with oligonucleotides. However papaverine has not this positive charge but a more flexible structure that theoretically made a priority for its fitting to the interacting molecules. In this observation papaverine has almost no interaction with G-quadruplex structure of telomeric sequences. The ΔT_m _ caused by papaverinewas calculated to about 5 °C indicating that papaverine does not intercalate F21T. We therefore conclude that the inhibition of telomerase is not due to intercalation of its substrate (Figure 5b). However, some reports indicated interaction of some papaverine derivatives with G-quadruplex sequences [21]. 

### 2.4. Papaverine Reduces Transcription Level of hTERT

It is already known that telomerase regulation is mainly due to the expression level of hTERT, the catalytic subunit of the enzyme, in spite of multiple possible points of regulation [22]. Therefore, mRNA levels of hTERT in control and treated cells were estimated using quantitative real-time RT-PCR technique. Using β2-microglobulin gene as a housekeeping gene, relative mRNA level of hTERT in 48 h treated HepG2 cells was strongly reduced so that in cells treated with 60 and 120 µM papaverine hTERT was estimated to about 50% and 45% of untreated controls, respectively (Figure 4b). Although its overall pattern is the same as the reduction of telomerase activity, the inhibition effect of papaverine in enzyme activity level is faintly stronger than down regulation of hTERT gene. The extent of telomerase inhibition by berberine, papaverine and (from our previous report in [17]) chelidonine in HepG-2 cells are more or less the same, however these alkaloids exhibit different levels of cytotoxicity and genotoxicity. Among these three alkaloids chelidonine is the strongest cytotoxic alkaloid, while berberine is a potent genotoxic compound. On the other hand, the observed down-regulation of hTERT by papaverine may well correlate with anti-HIV properties of this compound inhibiting HIV replication in H9 cell line and in peripheral blood mononuclear cell culture [11].

### 2.5. Papaverine Accelerates Senescence in HepG-2 Cells after Long Exposure to Very Low Concentrations

**Figure 6:**
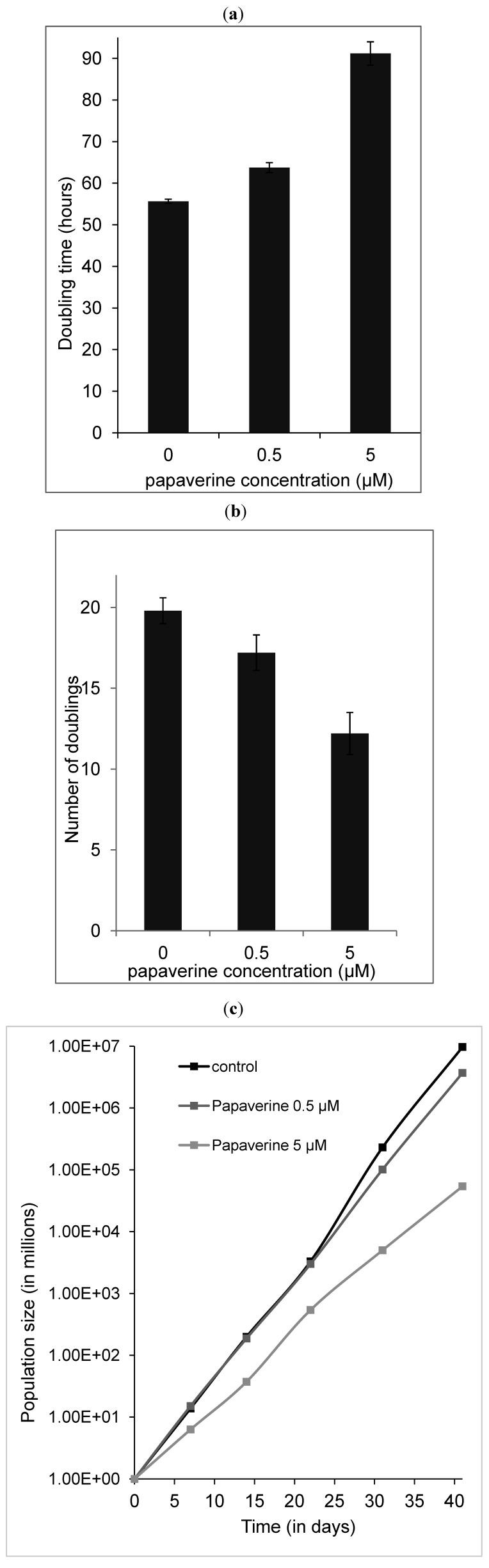
Influence of papaverine treatment on cell doubling time. (**a**) The doubling time, (**b**) number of doublings and (**c**) population size of untreated control HepG2 and treated cells with 0.5 and 5 µM papaverine after 41 days. Presented data are means ± SD of two independent experiments each in duplicates.

**Figure 7:**
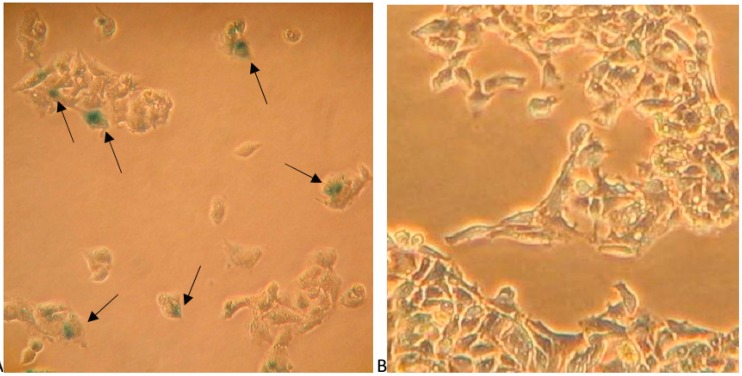
(**a**) β-Galactosidase staining of HepG2 cells treated with 5 µM papaverine for 48 h per passage after 41 days and (**b**) of untreated cells. Positively stained cells are marked by an arrow.

Long-term treatment of the cells with very low concentrations of papaverine increased doubling time in comparison with untreated cells. Continuous culturing HepG-2 cells in a concentration of 0.5 and 5 µM papaverine for 48 h per passage the doubling time of the cells increased to 63.7 ± 1.5 and 91.1 ± 1.3 h after 41 days respectively compared to 55.6 ± 0.9 h for untreated controls (Figure 6a). These concentrations were chosen as no sign of increase in cell death was detectable by trypane blue staining and visual morphology of the cells (Figure 7). The number of doublings in each series during this period is shown in Figure 6b. This mode of continuous treatment with small amounts of papaverine resulted in senescent cells that failed to be subcultured. Also the morphology of the cells changed: The treated cells appeared dark after beta-galactosidase staining and larger with high cytoplasmic to nucleus ratio, a characteristic typical for cell aging. Counting 1,000 cells in five random fields of each culture, positively stained cells in cultures that were continuously treated with 0.5 and 5 µM papaverine was estimated to 11.5% and 19.8% respectively in comparison with 1.9% in untreated control cells.

## 3. Experimental Section

### 3.1. Cell Culture and Cytotoxicity Test

HepG-2 cell line (ACC 180 from DSMZ, Germany) was maintained in 75 cm^2^ culture flasks in RPMI-1640 (Gibco, Invitrogen, Germany), supplemented with 10% heat-inactivated fetal bovine serum (PAA GmbH, Pasching, Austria), 100 U/mL penicillin, and 100 µg/mL streptomycin (PAA GmbH, Pasching, Austria) in 5% CO_2_ at 37 °C and 100% humidity. Cytotoxicity of papaverine hydrochloride (MW: 375.85, Sigma-Aldrich, Germany), was estimated using MTT (3-(4,5-dimethylthiazol-2-yl)-2,5-diphenyl-etrazolium bromide) [23] that purchased from Sigma-Aldrich.

Exponentially growing cells were seeded in 96 well plates with 10,000 cells per well. The medium was refreshed after 24 h while included different concentrations of papaverine. After 48 h treatment, the cells were washed with PBS and incubated 4 h in fresh medium including 0.5 mg/mL MTT. The reduced purple formazan product of MTT by living cells was dissolved in 200 µL of solvent (10% SDS and 1% acetic acid in dimethylsulfoxide) and measured by using a plate reader (BioTek, Winooski, VT, USA) at 570 nm. IC_50_ values of papaverine was calculated from dose-response curves of four logical repeats of the assay, each includes samples in triplicate.

To estimate the telomerase activity level in papaverine treated cells, subconfluent cultures were treated at three different concentrations; a relatively low concentration correlated with low cytotoxicity of papaverine, a second concentration was equal to IC_50_ to have the maximum possible treatment and still producing enough cells for analysis, and a third concentration represented the midpoint of the cytotoxicity curve. Treatment duration time was set to 48 h to have adequate time for cycling cells, because telomerase is active in a short period during S phase.

For long-term growth of HepG2 cells, papaverine treated or untreated cells were seeded into 75 cm tissue culture flask at 1 × 10^6^ for 4–5 days until 70%–80% confluency, then trypsinized and counted. Each time 1 × 10^6^ cells were subcultured into new flasks but in each passage the papaverine treated series were exposed to the desired concentrations of papaverine for 48 h and subsequently cells were fed with fresh normal medium. All the treatments were done in duplicate and the averages are presented in population analysis. 

### 3.2. Assay of Telomerase Activity

The cells after 48 h treatment with various concentrations of papaverine were washed twice with PBS and incubated in a lysis buffer containing 10 mM Tris-HCl, pH 7.5, 1 mM MgCl_2_, 1 mM EGTA, 0.1 mM phenylmethylsulfonylfluoride (PMSF), 5 mM β-mercaptoethanol, 0.5% CHAPS and 10% glycerol according to Kim *et al.* [24] on ice for 30 min and then centrifuged at 16,000 g for 30 min. Protein concentration of the supernatant was measured based on Bradford assay [25] using plate reader (BioTek) and analyzed with Gene5 software version 1.06. For each of control and/or treated cells 0.5 µg of extracted total protein was used in a quantitative real-time TRAP according to Hou *et al.* [26] with small modification. The reaction mix contained 0.1 µg each of primers TS (5'-AATCCGTCGAGCAGAGTT-3') and ACX [5'-GCGCGG(CTTACC)3CTAACC-3'] and 1× SYBR Green PCR Master Mix (GenetBio, Gobiz, South Korea). The reaction mixture was first incubated at 25 °C for 20 min to allow the telomerase in the protein extracts to elongate the TS primer by adding TTAGGG repeat sequences. q-TRAP assay was performed to compare telomerase activity in equal amounts of protein extracts from samples by real-time thermal cycler Rotor Gene 3000 (Corbett Research, Sydney, Australia) that was started at 95 °C for 10 min, followed by a 40-cycle amplification (95 °C for 20 s, 50 °C for 30 s, and 72 °C for 90 s). The threshold cycle values (Ct) were determined from amplification plots as analyzed with Rotor Gene 6.01 (log increase in fluorescence versus cycle number) and compared with standard curves generated from serially diluted cell lysate of untreated control samples [27]. Telomerase activity of cells treated with different concentrations of papaverine was determined in at least three logical repetitions. A known telomerase inhibitor with related structure, berberine [28] was used in parallel. In each experiment a negative control was included that consisted of a sample from untreated control HepG-2 cells in which telomerase was inactivated by boiling and/or RNase A treatment. The qTRAP product of cells treated with each concentration of papaverine was compared to those of untreated control cells, which was considered to have 100% activity.

### 3.3. RNA Isolation, Reverse Transcription and Real-Time RT-PCR

The cells were seeded in 12-well plate culture dishes, incubated with various concentrations of papaverine for 48 h. After harvesting the cells total RNA was isolated using RNeasy Mini Kit (Qiagen; Hilden, Germany) according to the manufacturer’s instruction. First strand cDNA synthesis was performed according to the protocol suggested for the Reverse Transcription System (AccuPower RT PreMix, Bioneer, South Korea, 1× Rotor Gene SYBR Green I Master Mix (Qiagen), 0.5 µM of each exon-spanning specific primer pairs for hTERT as previously described [27]. The expression level of each gene in each sample was normalized to that of β2-microglobulin, the housekeeping gene as internal control. Then the measurements compared to that of untreated control cells. The data collected from three repetitions including two measurements for each of the duplicated samples for the defined concentrations of the alkaloid.

### 3.4. FRET Analysis of Synthetic Double Labelled Telomeric Oligonucleotide

To estimate the potential binding of papaverine to telomeres, melting temperature of a synthetic double labelled oligonucleotide F21T 5'FAM-GGG(TTAGGG)_3_-3′TAMRA (Eurofins MWG Operon, Munich, Germany) was evaluated according to Mergny *et al.* [29,30] with small modifications. Briefly, the oligonucleotide was heated 10 min at 90 °C and quickly cooled on ice and aliquoted in 0.25 µM final concentration in a total volume of 25 µL in 0.1 M potassium chloride, 10 mM sodium cacodylate (pH 7.2) buffer and different amounts of the alkaloid to achieve the desired concentration. After 2 h incubation at 37 °C, the fluorescence intensity of samples was measured on a Rotor Gene 3,000 real-time thermal cycler (Qiagen) using filters suited for FAM by running a heating program starting from 37 °C to 95 °C by a rate of 0.5 °C/min. Melting temperature of samples comprising different concentrations of papaverine was compared with the control that contained no alkaloid.

### 3.5. β-Galactosidase Staining as a Senescence Marker

After removing medium, control and papaverine treated HepG-2 cells were washed in PBS, trypsinized, collected and counted. A small part of each sample was transferred to a 6-well plate. After 24 h the cells were fixed for 3–5 min at room temperature in 2% formaldehyde/0.2% glutaraldehyde, washed twice with PBS and incubated at 37 °C (without CO_2_) with fresh senescence-associated β-galactosidase (SA-β-gal) stain solution containing 0.04% X-gal, 40 mM citric acid/sodium phosphate, pH 6.0, 5 mM potassium ferrocyanide, 5 mM potassium ferrocyanide, 150 mM NaCl and 2 mM MgCl_2_ as described by Dimri *et al.* [31]. Staining was achieved in 4 h and positively stained cells (dark blue) were counted in at least 5 microscopic fields including almost 500 cells.

### 3.6. Statistical Analysis

Statistical analysis was performed by using standard student (t) test and a P < 0.05 was considered as the cutoff for significant difference.

## 4. Conclusions

In conclusion, the data from this study revealed a strong influence of papaverine on immortality of cancer cells. Telomerase activity in HepG-2 cells is inhibited by about 50% after 48 h treatment with 60 µM papaverine. While some synthetic derivatives of papaverine are known to have strong interaction with telomeric guanine-quadruplex structures [21], papaverine itself showed only a negligible ability for intercalation. However, it apparently stimulates several pathways resulting in growth arrest. Although stabilization of the folded state of telomere sequences along with down regulation of hTERT is a major mechanism in telomerase inhibition by berberine, multiple mechanisms may be involved in HepG-2 cell growth arrest by papaverine. It inhibits telomerase most likely by down-regulation of the hTERT gene while induction of cell senescence is obviously also involved. Since papaverine shows no genotoxicity (in contrast to berberine [32]) we suggest that this compound could functions as a valuable natural molecule to develop new drugs targeting immortality of cancer cells. Considering the currently clinical usage of papaverine as a vasodilator, our results illustrate the potential utility of papaverine, provided it can be chemically modified to an analogue that exhibits greater efficacy than what we reported here (probably in the nM level), exhibits bioavailability, metabolic stability and provides clinically acceptable pharmacokinetic and pharmacodynamic profiles to humans.
